# Retraction Note: MicroRNA-29 mediates anti-inflammatory effects and alleviation of allergic responses and symptoms in mice with allergic rhinitis

**DOI:** 10.1186/s13223-023-00807-1

**Published:** 2023-06-14

**Authors:** Jia Wang, Jinshu Yin, Hong Peng, Aizhu Liu

**Affiliations:** Department of Otolaryngology Head and Neck Surgery, Beijing Shijitan Hospital, Capital Medical University, No. 10 Yangfangdian Railway Hospital Road, Haidian District, Beijing, 100038 China

**Retraction Note: Allergy, Asthma & Clinical Immunology (2021) 17:24**

10.1186/s13223-021-00527-4

The Editor in Chief has retracted this article because of multiple overlaps between panels in Figs. 5a and c and 6d of the article and those in rows p-STAT3, HIF-1a and Cyclin D1 of Fig. 5 in a previously-published paper by different authors [1, now retracted]. The authors did not respond to the journal’s requests to send raw data and evidence that ethics approval had been obtained before the commencement of the study. The Editor in Chief has, therefore, lost confidence in the integrity of the article’s findings. The authors did not respond to any correspondence from the Editor about this retraction.
